# Engagement of health workers and peer educators from the National Adolescent Health Programme-Rashtriya Kishor Swasthya Karyakram during the COVID-19 pandemic: Findings from a situational analysis

**DOI:** 10.1371/journal.pone.0266758

**Published:** 2022-09-21

**Authors:** Monika Arora, Stefanie Dringus, Deepika Bahl, Zoya Rizvi, Heeya Maity, Smritima Lama, Amanda J. Mason-Jones, Deepak Kumar, Prairna Koul, Shalini Bassi

**Affiliations:** 1 Health Promotion Division, Public Health Foundation of India, Gurugram, Haryana, India; 2 Independent Global Health Consultant, London, England; 3 Ministry of Health and Family Welfare, Government of India, New Delhi, India; 4 Department of Health Sciences, University of York, Heslington, York, England; Imperial College London, UNITED KINGDOM

## Abstract

**Background:**

To understand the impact of COVID-19 on implementation of the peer education programme of the National Adolescent Health Programme-Rashtriya Kishor Swasthya Karyakram (RKSK); repurposing of the RKSK health workers and Peer Educators (PEs) in COVID-19 response activities and effect on adolescents´ health and development issues.

**Methods:**

Virtual in-depth interviews were conducted with stakeholders (n = 31) (aged 15 to 54 years) engaged in the implementation of the RKSK and peer education programme at state, district, block, and village levels in Madhya Pradesh and Maharashtra (India). These interviews were thematically coded and analysed to address the research objectives.

**Results:**

Despite most peer education programme activities being stopped, delayed, or disrupted during the pandemic and subsequent lockdown, some communication networks previously established, helped facilitate public health communication regarding COVID-19 and RKSK, between health workers, PEs, and adolescents. There was repurposing of RKSK health workers and PEs’ role towards COVID-19 response-related activities. PEs, with support from health workers, were involved in disseminating COVID-19 information, maintaining migrant and quarantine records, conducting household surveys for recording COVID-19 active cases and providing essential items (grocery, sanitary napkins, etc.) to communities and adolescents.

**Conclusion:**

PEs with support from community health workers are able to play a crucial role in meeting the needs of the communities during a pandemic. There is a need to further engage, involve and build the skills of PEs to support the health system. PEs can be encouraged by granting more visibility and incorporating their role more formally by paying them within the public health system in India.

## Introduction

The COVID-19 pandemic has presented numerous challenges to health systems globally [1]. India has implemented control measures similar to other countries to curtail the spread of COVID-19. This led to decision-makers making difficult choices, which involved prioritising preventive and curative COVID-19-related care while scaling back other areas of healthcare delivery, including but not limited to, adolescent health [2]. The central and state government continue implementing preventive public health measures [3, 4] including lockdowns, closure of educational institutions (schools, colleges), physical distancing, compulsory wearing of a mask, and restrictions on large gatherings and social events (deaths and marriages). However, the pandemic has also had severe direct and indirect consequences on the health outcomes of people and the health system functioning during India’s first lockdown (March-May, 2020). Health care services were disrupted for non-COVID-19 patient care due to the closure of routine outpatient departments, immunization clinics, antenatal services [5], and hospitals being designated as COVID-19 treatment centers [6]. Data from the National Health Mission highlighted a reduction in availing medical treatment (including both inpatients and outpatients) and emergencies for both infectious and non-communicable diseases (NCDs) during the first lockdown [7].

Implementation of several national health programmes, including India’s National Adolescent Health Programme-Rashtriya Kishor Swasthya Karyakram (NAHP-RKSK), were impacted during COVID-19. RKSK was launched across India in 2014, aiming to reach all adolescents, including both males and females, rural and urban, married and unmarried, and those in and out of school. RKSK encompasses various interventions involving multiple implementation stakeholders (Table 1). Interventions specified in Table 1 operate at school, community, and health facilities’ levels. The peer education programme, one of the core interventions under RKSK, is expected to increase adolescents’ engagement with health services and improve their knowledge, attitudes, and life skills in six thematic areas- mental health, injuries, and violence, sexual and reproductive health, NCDs, substance misuse, and nutrition.

**Table 1:** Overview of RKSK interventions and health workers [8, 9] involved in programme implementation.

Activities/ Services	Service Delivery	RKSK Stakeholders
**1. Facility-Based Intervention**		
Adolescent Friendly Health Clinic (AFHC)	A dedicated room for adolescents with the provision of clinical, counselling, and referral services. Commodities (Iron Folic Acid tablets and other medicines, pregnancy testing kits, sanitary napkins & contraceptives) are also disbursed to adolescents. AFHCs are operational at Primary Health Centers (PHCs), Community Health Centers (CHCs), and District Hospitals (DHs). AFHC at the DH level works as an Adolescent Friendly Health Resource Centre (AFHRC) responsible for capacity building of health care providers and act as a repository for RKSK IEC materials.	Medical Officers (MO), RKSK counsellors, Auxiliary Nurse Midwives (ANM), Specialists
**2. Community-Based Interventions**		
Adolescent Health & Wellness Days (AHWDs)	AHWD is organised quarterly at the village level to provide preventive and promotive interventions and increase awareness among adolescents, parents, families, and other community stakeholders about RKSK six thematic areas, AFHCs and helplines.	Accredited Social Health Activist (ASHA), ASHA Facilitators, Anganwadi worker (AWW), Peer Educators (PEs), ANM, MOs, Non-Governmental Organisation (NGO) Trainer/Mentor and Adolescent Health counsellors
Adolescent Friendly Club (AFC) Meetings	Meetings are organised once a month at the sub-centre (village level) under the guidance of ANM. Cover 5 villages/5000 population. Thus, 10–20 PEs from different villages are invited for these meetings. PEs clarify issues faced during their village-level PE sessions.	ANM, ASHA, ASHA Facilitators, PEs
Peer Education Programme	2 PEs (one boy and one girl) per village/1000population/ASHA habitation are selected to reach out to adolescents. Each PE forms a group of 15–20 boys or girls from the community and conducts weekly 1–2 hour participatory sessions using PE kits.	PEs, ASHA, ASHA facilitator, ANM, NGO mentor/ trainer, MO, Counsellors
Weekly Iron Folic Supplementation Programme (WIFS)	Screening of anaemia among girls and serving out-of-school adolescent girls in Anganwadi centres on a fixed day in a week to provide IFA tablets. Biannually Albendazole is provided for deworming.	ANM, ASHA, AWW
Menstrual Hygiene Scheme (MHS)	Girls are provided with knowledge and information regarding good menstrual hygiene and the safe disposal of sanitary napkins. ASHAs provide napkins at a subsidised rate of Rs. 6 per pack of 6 napkins to adolescent girls in schools and communities.	ASHAs
**3. School (government, government-aided municipal schools) based** [10] **interventions**		
School Health programme under Ayushman Bharat	The Government of India, under the Health and Wellness component of the Ayushman Bharat (Healthy India) Programme, intensified school-based health activities. These activities combine health education, promotion, disease prevention, and improved access to the health system at the school level. Two PEs/Saathiya, one boy and one girl support the health and wellness ambassador in carrying out the health promotion activities.	Trained School Teachers (Health & Wellness Ambassadors), Health and wellness messengers (2 students per section assist the health ambassadors), ANM, ASHA, AWW, Counsellors (school outreach), PEs (in-school & out of school)
Menstrual Hygiene Scheme (MHS)	Health education for adolescent girls and providing sanitary napkins and enabling other sanitation measures such as access to water and toilets in schools. Provide life skill courses to adolescents of class IX and XI.	School teachers
Weekly Iron Folic Acid Supplementation Programme (WIFS)	Screening of anaemia among adolescents and provide weekly IFA tablets to adolescents boys and girls and Albendazole biannually for deworming.	School teachers

The Community Health Workers in India like the ASHAs, ANMs, and AWWs have had to be diverted from their regular tasks to participate in the COVID-19 pandemic response. They were re-purposed to curb the increase in cases by participating in containment and response efforts such as contact tracing, community surveillance, and promotion of safe practices and to help decrease COVID-19 vaccine hesitancy among their communities [11–13]. These workers are closely working with PEs while implementing RKSK at the ground level. Thus, with this background, a situational analysis was conducted virtually as a part of the study to explore the implementation of the peer education programme of RKSK in two Indian states (Madhya Pradesh and Maharashtra) in 2020. Situational analysis aims to examine: a) Implementation of peer education programme during COVID-19, b) repurposing of the RKSK health workers and PEs i.e. in addition to their existing roles and responsibilities under the RKSK, their participation in COVID-19 response activities, c) effect of COVID-19 on adolescents´ health and development issues.

## Materials and methods

### Study setting and participants

The study was conducted in Madhya Pradesh and Maharashtra. Madhya Pradesh is a large state in central India with a population of 72.6 million and a literacy rate of 69.3%. Maharashtra, in India’s western peninsular region, is the second-most populous state with a population of 112.3 million and an 82.3% literacy rate [14]. Both states were among the most severely affected states in India due to COVID-19 [15, 16]. This trend remained from the start of the pandemic to its waning phase, with Maharashtra reporting an increasing number of cases despite the national lockdown [17]. Madhya Pradesh, one month into the pandemic, remained among the top five most impacted states with the highest number of fatalities [18]. In consultation with state health departments, two study districts were chosen from each state, i.e., Panna and Damoh from Madhya Pradesh and in Maharashtra, Nashik, and Yavatmal. The implementation status of the PE programme in the two districts i.e. a district where the PE programme had been implemented for a long time and the districts where the implementation had just started along with the demographic characteristics were considered while selecting the study districts [19, 20]. Study participants were recruited using a snowball sampling technique wherein the state RKSK team nominated participants from the district who then nominated participants from the block and further participants at the village level. Participants were sampled till no additional data was being reported by our study participants in context to the interview guide used for data collection to reach data saturation [21]. Participants who provided their consent were included in the study. Verbal consent was obtained from each participant as per the Indian Council of Medical Research’s revised guidelines during the COVID-19 pandemic [22]. For participants under the age of 18 years, informed consent was obtained from their parents followed by informed assent from the participants.

### Data collection

Data was collected through in-depth interviews (IDIs), using semi-structured interview guides tailored for each group of participants (n = 31). These in-depth interview guides were developed referring to RKSK Implementation Guidelines [8], Operational Framework [9], and consultations with the Central, State, and District RKSK officials. Interview guides were pilot tested to ensure content and face validity. The expert’s comments (n = 5) were received and appropriate modifications were made to ensure content validity. For face validity, the guides were pilot tested with the target group i.e. RKSK health workers (n = 5). An overview of respondents interviewed and the themes covered has been presented in S1 Table. These IDIs were conducted in Madhya Pradesh during (June-November, 2020) and Maharashtra (August-November 2020). IDIs were moderated by trained qualitative researchers accompanied by a note taker. Since data was collected virtually, building rapport with the respondents was imperative thus a, strategic approach was followed. This encompassed the introduction by the research team, explaining the study aims and objectives through Participant Information Sheet before commencing the data collection. Subsequently, during data collection, the interaction of the study team and the participants began with rapport-building questions like how long have you been living in this village, how long have you been in a particular role, their association with the health department, existing roles and responsibilities, etc. To mitigate the risk of COVID-19 transmission, virtual audio interviews using the Zoom platform and mobile phones were conducted. Most officials at the district level were interviewed on Zoom while those at the village level were interviewed over the mobile phone considering the technology empowerment. The average duration of each interview was 60–90 minutes. Interviews were conducted in English or Hindi and audio-recorded. Unique IDs were allocated to each participant by the research team. Interviews were translated and transcribed verbatim by members of the research team. Identifiable data was anonymised. Ethics approval for the research was obtained from the Institutional Ethics Committee of the Public Health Foundation of India (Reference # TRC-IEC-342.1/17).

### Data analysis

Each transcript was read several times by two authors independently, data were coded and organised thematically. Discrepancies between two authors in coding and organising data were discussed with a third senior author. The data analysis follows a sequential approach of deductive and inductive steps to derive themes and sub-themes [23]. In the end, five themes were generated to address the research objectives and these have been discussed in the results section.

## Results

### Demographic profile of study participants

In total, thirty-one participants (Females = 20, Males = 11) were interviewed from both study states representing the RKSK health workers at state, district, block, and village levels (Table 2). There were 18 participants from Madhya Pradesh and 13 from Maharashtra. The difference in the number of participants recruited by states and hierarchy (state, district, block, and village) has been attributed to the nominations provided by the contact person at a higher level (state and district). The participants’ ages ranged from 15 to 54 years and the affiliation period with RKSK ranged from 1 month to 5 years while the median affiliation period was 2 years (24 months).

**Table 2:** Study participants & their roles and responsibilities (N = 31).

S. No.	RKSK health workers	Roles & Responsibilities	Number of Participants (N)	
			Madhya Pradesh	Maharashtra
**State Level**				
1.	Nodal Officer	• Prepares adolescent Health Programme Implementation Plan (PIP) for the State		1
		• Implement RKSK at State/ District Level.		
		• Reviews records and submits PIP to a higher level		
**District Level**				
2.	Programme Manager /Chief Medical Officer (CMO)	• Appoints officer to supervise, monitor, and report on RKSK implementation	1	
		• Supervises, monitors, compiles, and reports on RKSK and NGO activities wherever applicable.		
3.	Community Mobilizer	• Supervises and monitors ASHAs and ASHA Facilitators of the district	1	
4.	Programme Coordinator	• Oversees RKSK implementation	1	
5.	District Reproductive and Child Health (RCH) Officer	• Provides training to MOs, ANMs, ASHAs, PEs, and Lady Health Visitors		1
		• Monitors and evaluates the programme		
6.	District Counsellors cum coordinator	• Provides clinical and counselling services at DH level AFHC		2
		• Coordinates outreach activities		
		• Record keeping & reporting		
		• Coordinates other RKSK programme activities at the block level		
7.	Counsellor at District Hospital	• Provides clinical and counselling services at DH level AFHC	1	
		• Coordinates outreach activities		
8.	Training Faculty (Faculty Training Centre)	• Monitors annual training plan implementation for different areas		1
		• Coordinates and plans activities at the training centre		
		• Develops training plans and conducts training of trainers (MOs & other paramedical staff)		
9.	NGO Representative	• Recruits staff to support PE sessions and AFC meetings	1	
		• Keep records and submits reports to higher levels		
**Block Level**				
10.	Medical Officer (Primary Health Centre)	• Dual responsibility of Medical Officer at PHC and AFHC (on outreach days)		1
		• Provide training of the selected PEs and ASHAs under RKSK.		
		• Monitor AHWDs & AFCs		
11.	CHC Counsellor	• Provides counselling to adolescents on RKSK’s six themes	1	
		• Supports district RKSK nodal officer to organize RKSK activities in their catchment area		
**Village Level**				
12.	ANMs	• Moderate monthly AFCs		2
		• Provide counselling services at PHC		
		• Organise AHWDs and provide group/individual sessions with parents on adolescent health needs		
		• Submit a monthly progress report to PHC medical officer		
13.	ASHA Facilitators	• Supervise up to 12 ASHAs	2	
		• Attend at least one PE session per month to resolve issues raised by ASHAs and/or PEs		
		• Collect and consolidate PE monthly reports		
14.	ASHAs	• Select & recruit PEs in consultation with NGO trainer-cum-mentor (where applicable)		2
		• Provide supportive supervision to PEs		
			5	
		• Attend village level PE sessions		
		• Collect and consolidate PE monthly reports		
		• Help organise and mobilise adolescents and other stakeholders for AHWD		
15.	NGO Mentor cum Trainer	• Appointed in states with NGO-led implementation models	1	
		• Select and recruit PEs along with ASHAs		
		• Provide supportive supervision to PEs		
		• Facilitate AFC meetings		
16.	AWW	• Mainly engages in WIFS programme	1	
		• Helps in organising AHWD and mobilising community participation		
17.	PEs	• Conduct sessions with adolescents at village level on RKSK’s six themes	3	3
		• Provide referrals for AFHCs and/or counselling		
		• Participate in AFCs and in AHWDs		
		• Maintain and prepare a PE diary and a monthly composite PE sessions’ report		

Key findings from this situational analysis are described under the following themes and sub-themes: 1) implementation of Peer Education programme during COVID-19; a) connectivity between RKSK health workers and PEs and between PEs and adolescents; b) effect of COVID-19 on adolescent friendly health services (AFHCs and helplines), and; 2) repurposing of the RKSK health workers and PEs; 3) adolescents’ health and development issues during COVID-19.

### Implementation of Peer Education programme

In the face of the pandemic, all activities related to the Peer Education programme implementation were either on hold, significantly delayed, or altered. Disruptions in the programme implementation were due to the restrictions put in place due to COVID-19 (lockdown, physical distancing, restricted movements, etc.). The stalled activities ranged from signing contracts with Non-Governmental Organisations (NGOs) to implement programme activities such as Adolescent Friendly Club (AFC) meetings, recruitment and training of PEs, delivery of PE sessions, and community outreach activities such as Adolescent Health and Wellness Days (AHWDs), school outreach activities. The District Coordinator from Madhya Pradesh said, “*due to COVID-19*, *the entire programme has been on pause*, *otherwise we would have received the copy of the agreement (between the state government and NGO) by now and started the work including staff selection*. *Due to a rise in the number of COVID-19 cases*, *we have PE training on hold”*. The District Reproductive and Child Health (RCH) officer in Maharashtra explained, “*We conduct outreach programmes in 5–6 schools every month but this year due to COVID-19*, *we haven’t been able to do it*”. The RKSK Nodal Officer in Maharashtra reiterated what the District level officials reported that to curb the spread of COVID-19, RKSK activities were on hold during the initial lockdown period.

*We have received guidelines from Government of India regarding COVID-19*. *According to those*, *from June we have to start some activities in areas other than the containment zone and the buffer zone*. *This includes activities like OPD (Out Patient) services in the clinics*, *holding AHWDs and Adolescent Friendly Club Meetings*, *while following all the guidelines provided by GoI and observing the situation in a particular area*. ***(RKSK Nodal Officer*, *Maharashtra*)**

In Maharashtra, the faculty at the District Level Training Center reported how the training of trainers for the RKSK programme was also impacted by the COVID-19 pandemic sharing *“Now*, *due to COVID some trainings are planned online and some are offline*.*”*

### Connectivity between RKSK health workers and PEs, and between PEs and adolescents during COVID-19 lockdown

Participants shared that PE sessions and information dissemination via in-person methods at the village level were put on hold due to COVID-19 restrictions. However, some communication, through mobile phones (via calls, WhatsApp, videos/ films) with PEs, related to COVID-19 and peer education programmes existed during the lockdown. Pre-existing WhatsApp groups for RKSK related activities, between ASHAs and PEs, were used for this communication. This informal and unplanned communication was evidence of the pivot, from the original guidelines [9] and shift from PE programme implementation pre-COVID to accommodate needs emerging due to the COVID context. This information was further shared with friends, family, and other adolescents.

*We are giving information to PEs through WhatsApp only*. *All information given is related to coronavirus, like what precautions they should take, frequent hand washing, wearing a mask when going out, cleaning used masks, and maintaining a distance of at least 6 feet*. ***(ASHA, Madhya Pradesh)***

In-person interaction with adolescents continued to occur during home visits by ASHAs and ASHA facilitators as part of the Weekly Iron Folic Acid Supplementation (WIFS) programme or other maternal and child health programmes.

*Ma’am we have home visits*. *I go with my ASHA to weigh new babies*, *or if there is a pregnant woman in the village then I have to go to at least one house with the ASHA*. *There are high chances that there are adolescents in that house*. *In those houses*, *I go and give out the iron tablets and tell them about menstruation etc*. ***(ASHA Facilitator*, *Madhya Pradesh*)**

Additionally, an NGO representative described how a trainer-cum-mentor, responsible for providing supportive supervision to PEs, had kept in touch with some PEs in Madhya Pradesh. He said, “*One mentor is in touch with 20 PEs (through telephone)*. *Since they (PEs) stay in the same village*, *they may talk to the adolescents one to one physically*, *or in small groups under the supervision of mentor over video call”*.

PE also reported sharing information about COVID-19 to other adolescents in their communities as instructed by ASHA:

*We meet girls in the coaching classes and we talk about wearing a mask and frequent hand washing*. *ASHA asked us to inform others about it*. ***(PE Female*, *Madhya Pradesh***)

Despite these successes of communication, some hindrances in the flow of information remained; for example, not all PEs could be reached via mobile phones due to lack of access. In many instances, there was sporadic and brief communication with those that were able to connect:

*There are some pockets in our district where there is no network*. *Some adolescents do not have their cell phones; some use their family member’s phones*, *so they may not be available to talk for that long when a mentor calls*. ***(NGO Representative*, *Madhya Pradesh*)**

### Effect of COVID-19 on adolescent health services (AFHCs and Helpline)

In both the states, participants reported adverse impacts on the delivery of health services, including AFHCs. During the initial lockdown period, while the District Coordinator and one of the counsellors from the AFHCs at Community Health Centres (CHC) in Madhya Pradesh reported that AFHCs remained closed with no tele-counselling provision. At this time, counsellors were involved in other COVID-19 related duties. As reported by an AFHC counsellor at a CHC in Madhya Pradesh “*…the clinic is closed nowadays*. *We have to check migrant labourers; whether have been screened or not for COVID-19*. *We collect data and send it to the concerned authorities*. *Nothing like that (tele-counselling etc*.*) is provided*.” In contrast, the counsellor from the district hospital in Madhya Pradesh mentioned providing tele-counselling services to adolescents.

*If I have numbers (contact no*.*) of the adolescents then I call them or those who have my number they call me*. *Yes*, *we are doing tele counselling*. ***(Counsellor*, *Madhya Pradesh*)**

The participants were also asked if there was any alternative available for adolescents such as a telephone helpline. There was a mixed response from the two study states participants. In Maharashtra, most of the participants were unaware of a helpline. A counsellor from Maharashtra mentioned using the 1098, a 24-hour emergency outreach service helpline for children (0–18 years) in distress, a service of the Ministry of Women and Child Development operating through Childline India Foundation [24, 25] and an active 104-helpline before COVID-19.

*Like for sexual abuse cases amongst teenagers I took help of 1098 helpline*. ***(Counsellor*, *Maharashtra*)**

In contrast, a PE in Maharashtra *“No we don’t have any helpline number for our village*.*”* explained that they were unaware of any helpline for adolescents.

Whereas, in Madhya Pradesh most participants were aware of the helpline and adolescents used the helpline to help solve their issues when they were unable to visit the AFHC or felt uncomfortable sharing their health issues/ problems with the Community Health Workers in their village. As explained by the counsellor, in Madhya Pradesh “*If adolescent cannot come or call us*, *they call helpline (104) and get information from them*.*”*

*Calling 104 was usually an effective way for adolescents to solve their problem when they may not feel comfortable speaking to an ASHA*, *as stay in the same village*. ***(ASHA*, *Madhya Pradesh*)**

However, the counsellor from CHC in Madhya Pradesh expressed concern about the inability to divert calls from 104, if adolescents require further or more individualised advice or medical care.

*Yes*, *we have*. *104 is the toll-free number*. *Lot of teenage girls and boys call the number and talk about their problem*. *No call (from 104) has been diverted to me*, *till date*. *We do not have that facility*. ***(CHC Counsellor*, *Madhya Pradesh*)**

After the reopening of AFHCs in both states, the footfall of adolescents was reported to have decreased significantly. This decrease may have been attributed, in part, to parents’ hesitation in sending their children to clinics due to fear of COVID-19 transmission as reported by an ANM in Maharashtra *“Currently due to lockdown and the COVID pandemic*, *adolescents are not coming*.*”* In Maharashtra, a decline in footfall may also have reduced clinics’ functional days or vice-versa.

*Before COVID*, *the clinic was regular (5 days a week)*. *These days the footfall is much lesser*, *only 6–7 adolescents come in a day compared to 20 adolescents in a day before the pandemic*. ***(Counsellor*, *District Hospital*, *Maharashtra*)**

Due to COVID-19 restrictions, there may also have been gender differences in those who were accessing the services in clinics. A counsellor from AFHC reported (Maharashtra), “*before COVID-19 there would be 30–32 adolescents in a day and there were more female visitors than males*. *But there is not much difference now during COVID*.”

### Repurposing of RKSK health workers and PEs to support COVID-19 response: During and post-lockdown

Several respondents reported repurposing in terms of roles and responsibilities to support COVID-19 response-related activities during and post COVID-19 lockdown. The PEs who have been trained under RKSK adapted to the context and undertook COVID-19 response activities by disseminating COVID-19 information to community members including adolescents. With the intent of tracing and tracking, they helped maintain migration and quarantine records of people migrating from urban cities to villages, and records were submitted at the block level. AWWs, though under the aegis of the Ministry of Women and Child Development, also supported the ASHAs and ANMs by providing door-to-door screening services during the beginning of the COVID-19 pandemic.

*Yes*, *ma’am we have to make door-to-door visits to ask if anyone had any symptoms- like fever*, *cold*, *cough etc*. *I used to go with the ANM and the ASHA to every house and ask them if they had any symptoms*. *No ma’am wherever the ANM did not go we would go there*. ***(AWW*, *Madhya Pradesh*)**

In Maharashtra, RKSK counsellors and counsellors of other health programs like Integrated Counselling and Testing Centers (ICTC) under the National AIDS Control Programme reported providing COVID-19 related counselling at help desks set up at out-patient departments and the in-patient department wards of District Hospitals. Similarly, from Madhya Pradesh, a counsellor from CHC reported his involvement in checking the migrant labourers for COVID-19 symptoms.

*We have to check migrant labourers; whether they have been screened or not for COVID-19*. *(****CHC Counsellor*, *Madhya Pradesh***)

ASHAs in Maharashtra were tasked with conducting village-level household surveys for the “My Village, My Responsibility” [26] campaign initiated by the Government of Maharashtra. The campaign aims to survey and screen households to detect COVID-19 patients and those with other pre-existing conditions (diabetes, cancer, and hypertension). A few ASHAs from Maharashtra also reported making and distributing masks using their resources (money, fabric, sewing machine) for community members at no charge. ASHA Facilitators and ASHAs were also tasked with visiting and surveying households to monitor COVID-19 active cases.

Without any additional formal training, PEs also contributed during COVID-19. They helped ASHAs in sensitising villagers about COVID-19 information including physical distancing, compulsory wearing of masks, sanitization, and handwashing. It was reported by the NGO trainer-cum-mentor (Madhya Pradesh) “*(PEs) are distributing masks*, *preparing migrant workers’ lists who are coming back and maintaining information about the people who are quarantined*. *PEs also helped ASHAs paint walls with information regarding COVID-19 preventive and protective measures*. *This included information about social distancing*, *masks*, *and sanitization and handwashing*.*”*

In Maharashtra, PEs reported helping ASHAs by putting stickers as part of the “My Village, My Responsibility” [26] campaign.

*As part of the My Village*, *My Responsibility campaign*, *we helped ASHA to put stickers outside the houses in the whole village*. *On that sticker*, *we write the number of family members*, *with their age*, *and if anyone has any comorbidities like diabetes*, *and if any member has a fever*. ***(PE Male*, *Maharashtra*)**

PEs also provided essential items such as grocery and menstrual hygiene products to adolescents and/or their families in the villages living in containment (*only essential activities allowed with strict perimeter control ensuring no movement of people*) or red zones (*areas with high COVID-19 caseload*).

*Those who were in red zones and not able to go out and buy anything*. *So*, *they call us (PEs) for necessities like milk*, *bread*, *and even medicines and we buy them and leave them outside their home*. ***(Male PE*, *Maharashtra*)**

Just like the ASHAs, AWWs, and ASHA Facilitators, the ANMs in Maharashtra were also given additional duties during the COVID-19 pandemic, including conducting door-to-door screening surveys.

*I did some surveys when some areas were declared as containment zones in the villages*. ***(ANM*, *Maharashtra*)**

### Adolescents´ health and development issues during COVID-19

There was limited and mixed data on the potential effects of COVID-19 on adolescents’ health and development issues. Participants from both states shared that adolescents faced challenges pertaining to education due to COVID-19 related school closures.

*Their online classes are happening*, *and a lot of children are saying we don’t understand much in these online classes*. *I think in face-to-face teaching; they understand quickly in comparison to online classes*. *In our village*, *online study content is received on WhatsApp*. *According to adolescents*, *the information is so long that they lose interest in reading the text*. ***(ASHA*, *Madhya Pradesh*)**

Few participants reported that the adolescents did not face severe challenges like mental health or sexual abuse during the pandemic. PE also highlighted that mental health was the most discussed topic during PE sessions with adolescents at the village level before COVID-19.

*Adolescents are wearing masks and meeting their friends*, *so I don’t think that COVID-19 is affecting their mental health*. ***(ASHA*, *Madhya Pradesh*)**

*The same number of cases of sexual abuse had come*, *both before and after COVID-19*, *only 2–4 cases came each month*. ***(Counsellor-District Hospital*, *Maharashtra*)**

*Mental health is the most discussed topic among adolescents as I think they are more stressed*, *so they like sharing that with us*. ***(PE*, *Maharashtra*)**

PEs also reported that COVID-19 appeared to have had some effect on menstrual health hygiene. Two female PEs (Maharashtra) explained, *“Sanitary pads/ napkins were not easily available*. *We contacted ASHA and ASHAs provided pads to the girls who were having difficulties in procuring them*.” There appeared to be a lack of demand in some places even pre-COVID, as reported by NGO trainer/mentor (Madhya Pradesh), “*they very rarely use pads in the villages*. *Our NGO used to sell pads which cost rupees 15(USD 0*.*20)*. *Few bought but most said that they feel better using cloth*.*”*

## Discussion

COVID-19, an unprecedented global health emergency, resulted in the disruption of healthcare delivery to people of all age groups, including adolescents. Literature suggests several consequences of COVID-19 and one of the most impacted groups would be the adolescents due to school closures, increased unemployment, etc. putting them at an increased risk of dropping out of school, gender gaps in education, mental health conditions, early marriage, smartphone addiction, etc. [27]. To the best of our knowledge, this is the first study in India that has attempted to understand what happened to the national adolescent health programme (NAHP-RKSK) at the time of COVID-19 and how PEs without any additional formal training, along with the health workers, played a crucial role in the COVID-19 response-related activities in the community of selected districts of Madhya Pradesh and Maharashtra.

### Implementation of Peer Education programme

Findings from our study indicate that RKSK and its core interventions were put on hold or altered as efforts were diverted to COVID-19 response activities. Other countries [28] have also reported the diversion of their health systems to solely focus on COVID-19. RKSK and its core interventions aim to address adolescent health issues and if such interventions are hampered, it can affect development gains made in the past few decades [29] and negate improvements gained from previous maternal, child health, and nutrition-related national programmes [30]. For instance, disruption due to COVID-19 on programmes to end child marriage, coupled with the pandemic-caused economic downturn, could result in 13 million more child marriages over the next decade (2020–2030) across the globe [31].

Evidence from previous pandemics in sub‐Saharan Africa and recent evidence from Ghana, Nigeria, and Kenya has revealed possible disparaging mid-and long‐term impacts of COVID‐19 on adolescents [28]. Hence, in India, RKSK has a significant role to play in ensuring that developmental gains made in the area of adolescent health (in the field of nutrition (anaemia), SRH (teenage marriage, menstrual hygiene, use of contraceptives), violence (gender-based violence), etc.) are not reduced or lost.

### Connectivity between RKSK health workers and PEs, and between PEs and adolescents during COVID-19 lockdown

In some instances, despite lockdown, RKSK health workers continued to provide informal support and supervision to PEs and adolescents by communicating about RKSK and COVID-19 precautions through phone calls, WhatsApp, or home visits. But there existed a digital divide in connecting with all PEs due to a lack of mobile phones and/ or weak mobile networks. Some PEs maintained connections and support with adolescents in the community, either one-to-one or through video calls. At times group calls with mentors and PEs were organised in Madhya Pradesh. These findings indicate that pre-established communication networks played a critical role in keeping adolescents, PEs, and RKSK workers connected, but also highlight the existence of a digital divide in most low-middle income countries which has been brought to the forefront due to the pandemic [32]. Further, there was evidence of the vital link that PEs play between community and health workers as iterated in the programme guidelines [9].

### Effect of COVID-19 on adolescent health services (AFHCs & Helpline)

Our study data suggests access to health services was affected by the pandemic resulting in the closure of AFHCs, or reduction in clinic operating days, and diversion of the health workers to other COVID-19 related duties. A study reports that ASHAs’ and ANMs’ involvement in COVID-19 related response activities hampered the regular benefits ensured by RKSK such as the implementation of WIFS providing supplementary nutrition and provision of sanitary napkins [27]. However, our findings indicate that ASHAs and ASHA facilitators as part of WIFS and maternal and child health programmes were making house visits and were talking to adolescents regarding the Peer Education programme. It may be said that despite the pandemic, some efforts continued to reach out to adolescents. Moreover, we need these efforts to be sustained with clear job aids so that every adolescent in a village in India irrespective of location is benefitted uniformly.

As per our study, Madhya Pradesh already had a 104 toll-free helpline number that was widely used by adolescents before COVID-19. The Central Government of India introduced a free helpline (08046110007) to address psycho-social concerns due to COVID-19 [33] but the same was not reported from either of the two study states, despite data collection beginning a few months after the introduction of the helpline. Literature encourages greater accessibility to teleconsultations and online psychotherapy with national helplines catering to mental health needs [34]. These will be helpful during both the ongoing and future pandemics, helping adolescents to remain connected with services due to physical inaccessibility. Thus, COVID-19 has brought forward previously unrecognised healthcare service needs such as teleconsultations and toll-free helplines [35].

### Repurposing of RKSK health workers and PEs to support COVID-19 response

There was a repurposing of the RKSK health workers and PEs towards COVID-19 response activities such as disseminating COVID-19 information, some instances of providing sanitary napkins, delivering essential groceries to the community, making and distributing masks, maintaining migrant and quarantine records, etc. These tasks were carried out despite challenges such as increased COVID-19 caseloads in both states, the absence of additional PE training, and no additional financial support to PEs. These are indicative of the fact that, despite the unpreparedness, the RKSK health workers and PEs were able to actively respond to the pandemic situation and provided help in whichever manner possible. Our findings align with the global response. The ASHA workers were even facilitated by the Word Health Organization at the 75^th^ World Health Assembly and were awarded the “Global Health Leaders Award” for their crucial role in linking community during COVID-19 pandemic [36, 37].

### Adolescents’ health and development issues during COVID-19

In our study states, RKSK implementers interviewed perceived that there was no change/negative effect on the adolescents’ mental health. This could be attributed to various factors like the informal communication of health workers with PEs and adolescents, and mental health is the most commonly discussed theme during the PE sessions pre-COVID-19. Hence, adolescents may have been equipped with coping skills to manage the stress and anxiety due to pandemic. On the other side, our situational analysis began during the initial phases of the COVID-19 pandemic and a truer picture would arise later due to the prolonged nature of the pandemic. Moreover, the literature suggests COVID-19 could have a deleterious impact on the mental health of children and adolescents [38] and this may be due to a variety of reasons, varying from a low understanding of the entire situation, disruption in their regular schedule with school closures [39], problems of substance misuse, and heightened physical abuse (domestic violence, intimate partner violence) [40]. These topics fall under the six RKSK themes and there is plenty of information available about the same in different resources such as the PE training manual, PE Activity Book, and Reference Book of Frequently Asked Questions [41].

India is already dealing with poor mental health care infrastructure and the added stress of the pandemic has overwhelmed the health care workforce with increasing pressure on the public health infrastructure [30]. Studies discussed above indicate the need to focus on mental health as an upcoming health issue for both adolescent health programmes and in the larger context of public health. Therefore, PE interventions can be game-changers for the future of our country as they not only increase awareness but also encourage adolescents’ engagement as being beneficial for the overall health and well-being of adolescents [42].

Keeping in mind the growing need for mental healthcare in India [34] it may be suggested that the PEs as health navigators may help in providing support to the health system as a low-cost intervention as part of RKSK. Studies are indicating that in comparison to traditional health care providers, PEs are more cost-effective and may help overcome shortages of human resources since community health workers are also overburdened [43, 44].

Future program planning can be informed by our study findings to adopt a mix of high tech (digital platforms and tools), low tech (SMS and phone calls), as well as no-tech (community, teachers, and parents’ groups) approaches to reach more adolescents including PEs in a variety of contexts [32] while also assisting in overcoming the impact of future pandemics [45]. Looking at the current context in India, PE sessions can inculcate the importance of physical distancing, general hygiene, and other infection control measures vital in preventing the transmission of viruses. Our study suggests that PEs can be important stakeholders during a health disaster-related response, or a quick onset crisis, and in overworked resource-constrained health systems, especially in low-and middle-income countries like India. Hence, to keep the momentum and engagement of the PEs, there is a need to enhance PE’s skills through booster training, supportive supervision, granting them more visibility, and incorporating their new roles and responsibilities more formally within the health system [27]. The PEs could also be provided experience certificates or some remuneration in the form of stipends for their work to acknowledge their contribution and for PEs to get a sense of accomplishment. This also empowers them at the community level and makes them responsible citizens within the health system [46].

Despite the novel findings presented in this paper, a few study limitations are worth considering. All interviews were audio-recorded thus participants’ non-verbal cues could not be captured, which could have enriched descriptions of experiences and situations in the study. Due to snowball sampling, the number of participants at state, district, and village levels were skewed. Thus, there could be reporting bias by the participants due to their difference in the level of engagement with programme implementation. In context to the effect of COVID-19 on adolescent health and development issues, we have captured the perspective of various stakeholders other than PEs. We could not include adolescents who are part of RKSK in this study, thus the beneficiary perspective is missing. As indicated by the stakeholder, there was a limited impact so we did not explore these issues further by gender, socio-economic status, marital status, etc. Finally, our study was limited to a few participants from two states, limiting our findings’ generalizability. This study was conducted as part of a situational analysis to gather relevant information from different levels of stakeholders. The objective of the situational analysis was to finalize the research questions with context to COVID-19. The findings from this study helped in the development of the study tools for the main study. All the above limitations will be considered while conducting an ongoing larger study with a bigger set of participants in the same study sites.

## Conclusion

Our findings indicate that there were some avenues of access to health care available to the adolescents despite the pandemic. They also highlight how RKSK health workers and PEs who were already working in the community were repurposed to engage with the COVID-19 response to not only the adolescents but also their communities as a whole. PEs along with the health workers navigated this challenging time and demonstrated their resilience in the face of an emergency. While their roles may have been very critical in responding to the pandemic, some additional support in the form of training, compensation, etc. could help strengthen this model for the future.

## Supporting information

S1 Table An overview of respondents interviewed and themes covered. (DOCX)
