# Supplementary material for: Engagement of health workers and peer educators from the National Adolescent Health Programme-Rashtriya Kishor Swasthya Karyakram during the COVID-19 pandemic: Findings from a situational analysis
Source: PLoS One. 2022 Sep 21;17(9):e0266758. doi: 10.1371/journal.pone.0266758 (PMC9491530; doi:10.1371/journal.pone.0266758)
Supplement: S1 Table — (DOCX) [file pone.0266758.s001.docx]

**Table S1: An overview of respondents interviewed and themes covered**

|  | | **Respondents** | | | | | | | | | | |
| --- | --- | --- | --- | --- | --- | --- | --- | --- | --- | --- | --- | --- |
| **Themes** | | **State Nodal Officer** | **District Coordinator** | **Representative from NGO implementing the programme** | **NGO Mentor and Trainer** | **Medical Officer** | **Faculty Training Center** | **Counsellor** | **ANM** | **ASHA Facilitator** | **ASHA** | **Peer Educator** |
|  | **Research Objective 1: To understand the impact of COVID-19 on implementation of the peer education programme** | | | | | | | | | | | |
| Implementation and Coverage of the RKSK Programmme during COVID-19 | | **✓** | **✓** | **✓** | **✓** |  | **✓** | **✓** | **✓** | **✓** | **✓** |  |
| Impact of COVID-19 on implementation of PE programme | | **✓** | **✓** | **✓** | **✓** | **✓** | **✓** | **✓** | **✓** | **✓** | **✓** |  |
| Adolescent Friendly Health Clinics (AFHCs) and impact of COVID-19 on services | | **✓** | **✓** | **✓** | **✓** | **✓** |  | **✓** | **✓** | **✓** | **✓** | **✓** |
|  | **Research Objective 2: Repurposing of the RKSK health workers and Peer Educators (PE) in COVID-19 response activities** | | | | | | | | | | | |
| Responsibilities and engagement with Peer educator programme during COVID-19 | | **✓** | **✓** | **✓** | **✓** | **✓** | **✓** | **✓** | **✓** | **✓** | **✓** | **✓** |
| Engagement of PEs during COVID-19 response activities | |  | **✓** | **✓** | **✓** |  |  | **✓** | **✓** | **✓** | **✓** | **✓** |
| Engagement of state, district, block and village level stakeholders during COVID-19 response activities | | **✓** | **✓** | **✓** | **✓** | **✓** |  | **✓** | **✓** | **✓** | **✓** | **✓** |
|  | **Research Objective 3: Effect of COVID-19 on adolescent health and development issues** | | | | | | | | | | | |
| Adolescent health and development issues during COVID-19 | |  | **✓** |  | **✓** | **✓** |  | **✓** | **✓** | **✓** | **✓** | **✓** |
